# Continuous Cough Monitoring Using Ambient Sound Recording During Convalescence from a COPD Exacerbation

**DOI:** 10.1007/s00408-017-9996-2

**Published:** 2017-03-28

**Authors:** Michael G. Crooks, Albertus den Brinker, Yvette Hayman, James D. Williamson, Andrew Innes, Caroline E. Wright, Peter Hill, Alyn H. Morice

**Affiliations:** 1 Department of Academic Respiratory Medicine, Centre for Cardiovascular and Metabolic Research, Hull York Medical School, Castle Hill Hospital, Cottingham, HU16 5JQ UK; 2 Philips Research, Eindhoven, The Netherlands; 3 Philips Respironics, Pittsburgh, PA USA

**Keywords:** Chronic obstructive pulmonary disease, Cough, Disease exacerbation, Outpatient monitoring, Telemedicine

## Abstract

**Purpose:**

Cough is common in chronic obstructive pulmonary disease (COPD) and is associated with frequent exacerbations and increased mortality. Cough increases during acute exacerbations (AE-COPD), representing a possible metric of clinical deterioration. Conventional cough monitors accurately report cough counts over short time periods. We describe a novel monitoring system which we used to record cough continuously for up to 45 days during AE-COPD convalescence.

**Methods:**

This is a longitudinal, observational study of cough monitoring in AE-COPD patients discharged from a single teaching hospital. Ambient sound was recorded from two sites in the domestic environment and analysed using novel cough classifier software. For comparison, the validated hybrid HACC/LCM cough monitoring system was used on days 1, 5, 20 and 45. Patients were asked to record symptoms daily using diaries.

**Results:**

Cough monitoring data were available for 16 subjects with a total of 568 monitored days. Daily cough count fell significantly from mean ± SEM 272.7 ± 54.5 on day 1 to 110.9 ± 26.3 on day 9 (*p* < 0.01) before plateauing. The absolute cough count detected by the continuous monitoring system was significantly lower than detected by the hybrid HACC/LCM system but normalised counts strongly correlated (*r* = 0.88, *p* < 0.01) demonstrating an ability to detect trends. Objective cough count and subjective cough scores modestly correlated (*r* = 0.46).

**Conclusions:**

Cough frequency declines significantly following AE-COPD and the reducing trend can be detected using continuous ambient sound recording and novel cough classifier software. Objective measurement of cough frequency has the potential to enhance our ability to monitor the clinical state in patients with COPD.

## Introduction

Chronic obstructive pulmonary disease (COPD) is a common, progressive and debilitating respiratory disease and is estimated to become the third leading cause of death world wide by 2030. Acute worsening of symptoms including shortness of breath and cough are termed acute exacerbations (AE-COPD). AE-COPD often precipitate hospital admission and are a source of significant morbidity and mortality [1]. Exacerbation prevention and amelioration is thus one of the primary aims of COPD treatment.

Cough and sputum production are reported by between 60 and 80% of patients with COPD [2, 3], and chronic cough and mucus hypersecretion are associated with faster lung function decline, increased exacerbation rate and increased mortality in COPD. Cough is therefore an important feature in COPD in terms of risk stratification and prognostication; however, the impact of this symptom is underappreciated by clinicians [4]. Patients report increased cough preceding an AE-COPD suggesting that it may be an attractive option for disease monitoring, particularly as these data can be collected passively, without patient input [5, 6].

In light of the significant burden of disease associated with COPD, novel strategies have been developed with the aim of improving patient management. The use of technology to monitor patients in their homes (telemonitoring) is one of these strategies. However, there is no compelling evidence that current telemonitoring methods in COPD are capable of identifying clinical deterioration early or preventing adverse outcomes including exacerbations requiring hospital admission or death [7–9]. A possible explanation for this is the inability of current monitoring strategies to accurately identify early clinical deterioration [7]. This may in part be due to the lack of useful objective physiological biomarkers of disease activity. Remote cough monitoring has potential to change this. However, existing cough monitors are generally worn devices that are designed to monitor short time periods. We have previously reported the ability of a hybrid cough monitoring system consisting of the hull automated cough counter (HACC) and Leicester Cough Monitor software (LCM) to detect a significant fall in cough frequency following AE-COPD when measured on days 1, 5, 20 and 45 [10]. However, to turn these observations into a practical monitor for the early detection of symptom worsening before exacerbation, continuous real-time analysis of cough frequency is required. Here we report the results of a cough monitoring system that records ambient sound using remote microphones and analyses cough frequency using a novel semi-automated cough classifier. This passive monitoring requires no patient compliance but is limited to those who are predominantly housebound. We validate this system against the hybrid HACC/LCM system and explore its capability to monitor daily cough frequency and detect meaningful change in the domestic environment.

## Methods

### Study Design and Subject Selection

This is a longitudinal, observational study of continuous remote cough monitoring using a novel cough monitoring system during AE-COPD convalescence. Inclusion criteria have been reported in brief previously [10]. Consecutive patients admitted to a single teaching hospital with an AE-COPD associated with increased cough and one more of breathlessness, increased sputum volume, or purulence were recruited to the study. Inclusion criteria stated that subjects should have a pre-existing diagnosis of COPD, be aged between 40 and 80 years and have a 10 or more pack-years of smoking history. Subjects were excluded if they had experienced an AE-COPD within the preceding 8 weeks or if they had clinically significant or unstable concurrent disease. The study protocol was approved by the local research ethics committee (LREC Number 11/NW/0643 and R&D Number R1229).

### Study Protocol

Continuous cough monitoring was undertaken in the patient’s home for 45 days following hospital discharge. The continuous monitoring system was composed of two laptop computers (Dell E6410, Dell, TX, USA) with attached microphones. Systems were positioned in the patients’ bedroom and a second room where the patient reported spending most of their time during the day. Audio captured by the remote microphones was stored on the laptop computer and subsequently analysed using bespoke semi-automated cough classifier software.

**Fig. 1:**
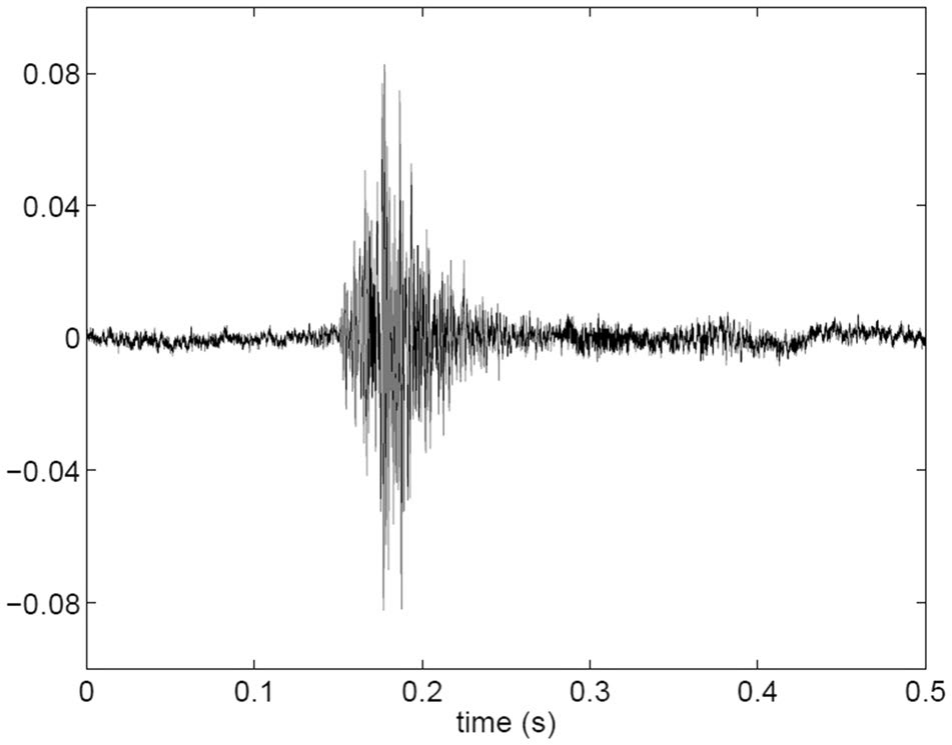
An example of a single cough recorded in the bedroom of patient 12. The audio sampling rate was 22,050 Hz

The bespoke cough classifier software works by extracting audio features from the full audio stream. The audio features are both temporal (e.g. total energy) and spectral (e.g. spectral tilt) where the latter is obtained using a frequency transformation on a perceptually relevant frequency scale. The cough classifier software was trained using a semi-supervised machine learning approach where a relatively short epoch of data was annotated by a member of the study team (1–2 days audio data contained an adequate number of coughs for classifier training). This was performed for each participant and site as different rooms have unique acoustic signatures. The training process required 3–4 h human involvement per subject. A representative illustration of the audio features of a single cough event is presented in Fig. 1. The trained classifier software was then used to detect the coughs in the entire audio stream (up to 45 days). The number of detected cough events was reported over 24 h (midnight to midnight). The sensitivity of the classifier software was deliberately minimised in favour of high specificity and thus positive predictive value. This approach was adopted to optimise trend analysis.

On days 1, 5, 20 and 45 following hospital discharge, the patients underwent additional monitoring using a validated, hybrid cough monitoring system composed of the HACC and LCM software as previously described [10]. The cough counts obtained were compared with those obtained during the same time period using the continuous monitoring system with the aim of evaluating the consistency of sensitivity and therefore the ability to detect trends.

In addition to cough monitoring, subjects completed daily diary cards reporting symptoms of shortness of breath, cough, sputum colour, chest tightness and sleep disturbance using a 5-point Likert scale. On days 1, 5, 20 and 45, the subjects also undertook home FEV1 monitoring using a hand-held spirometer (Vitallograph, UK) and completed the Leicester Cough Questionnaire (LCQ), COPD Assessment Test (CAT) and Hull Airways Reflux Questionnaire (HARQ). The results of these latter assessments have been reported previously [10].

### Data Analysis

#### Continuous Cough Monitoring

For each detected cough, a time stamp was created. Time stamps were counted over a full day denoted by *d*, with *d* = *0* identifying the day of discharge. With the purpose of detecting trends, these counts were smoothed over time using the mean data over the prior and subsequent 2 days. This creates a delay of 2 days in the processing chain. The resulting smoothed value is denoted as *C(d)*.

### Correlation Between Continuous Cough Monitoring, the HACC/LCM Cough Monitor and Symptoms

The cough counts obtained by the continuous monitoring system and the hybrid HACC/LCM system were mean normalised to correct for the different sensitivity of the two systems. The correlation was estimated using Pearson’s correlation coefficient (r). The difference in absolute cough count between HACC/LCM and the continuous cough monitoring system was analysed using the Mann–Whitney *U* test. The change in cough frequency and subjective cough scores were analysed using repeated measures ANOVA. For the purposes of this latter analysis, missing data were imputed as the subsequent available measurement. In the event of no subsequent measurements being available, the last available measurement was used. Pearson’s correlation coefficient was used to assess the correlation between cough count, symptoms scores, quality-of-life questionnaires and home FEV1 measurement.

## Results

A total of 24 subjects were screened with 19 subjects entering the study. One subject withdrew prior to commencing monitoring and no data were available for two subjects due to device failure (Table 1).

**Table 1:** Subject characteristics

Demographic	Subjects (%)
Number	*n* = 18
Age (mean ± SD)	68.1 ± 7.3
Gender	
Male	8 (44.4)
Female	10 (66.6)
FEV1 l/min (mean ± SD)	0.99 ± 0.53
COPD treatment^a^	
SABA	15
SAMA	1
LABA	2
LAMA	13
ICS	0
LABA/ICS	14
Smoking status	
Current smoker	7 (38.9)
Ex-smoker	11 (61.1)

*SABA* short-acting beta-2 agonist, *SAMA* short-acting muscarinic antagonist, *LABA* long-acting beta-2 agonist, *LAMA* long-acting muscarinic antagonist, *ICS* inhaled corticosteroid

^a^Information missing for 1 patient

Continuous cough monitoring data were available for 16 subjects with a total of 568 monitored days. HACC/LCM monitoring data undertaken on days 1, 5, 20 and 45 were available for 18 subjects with a total of 63 monitored days. Time-aligned cough monitoring data from both HACC/LCM and the continuous cough monitoring system were available for 47 monitored days (16 subjects). Paired continuous cough monitoring data and symptom scores were available for 14 subjects (45 monitored days). The most common reason for missing data was device failure resulting from loss of power. Patient readmission to hospital was another reason for missing data.

### Continuous Cough Monitoring

**Fig. 2:**
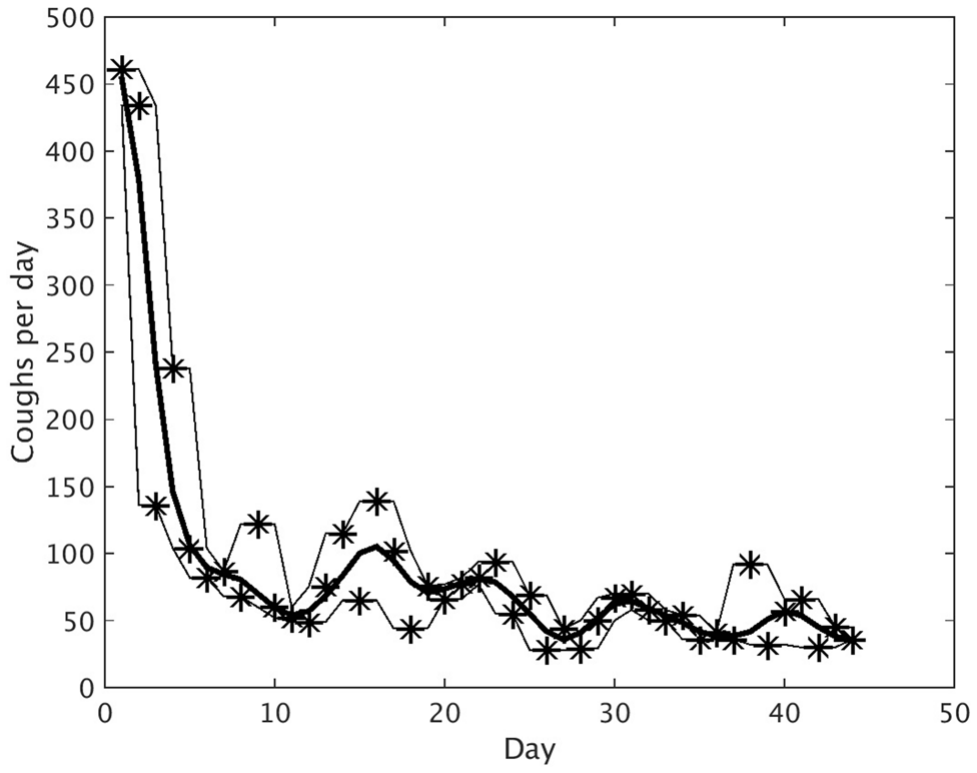
Graph representing the actual daily cough count (*stars*) and smoothed cough count (*thick line*) for an individual subject during AE-COPD convalescence. The *thin lines* represent the maximum and minimum of the actual daily cough count over 3 consecutive days

**Fig. 3:**
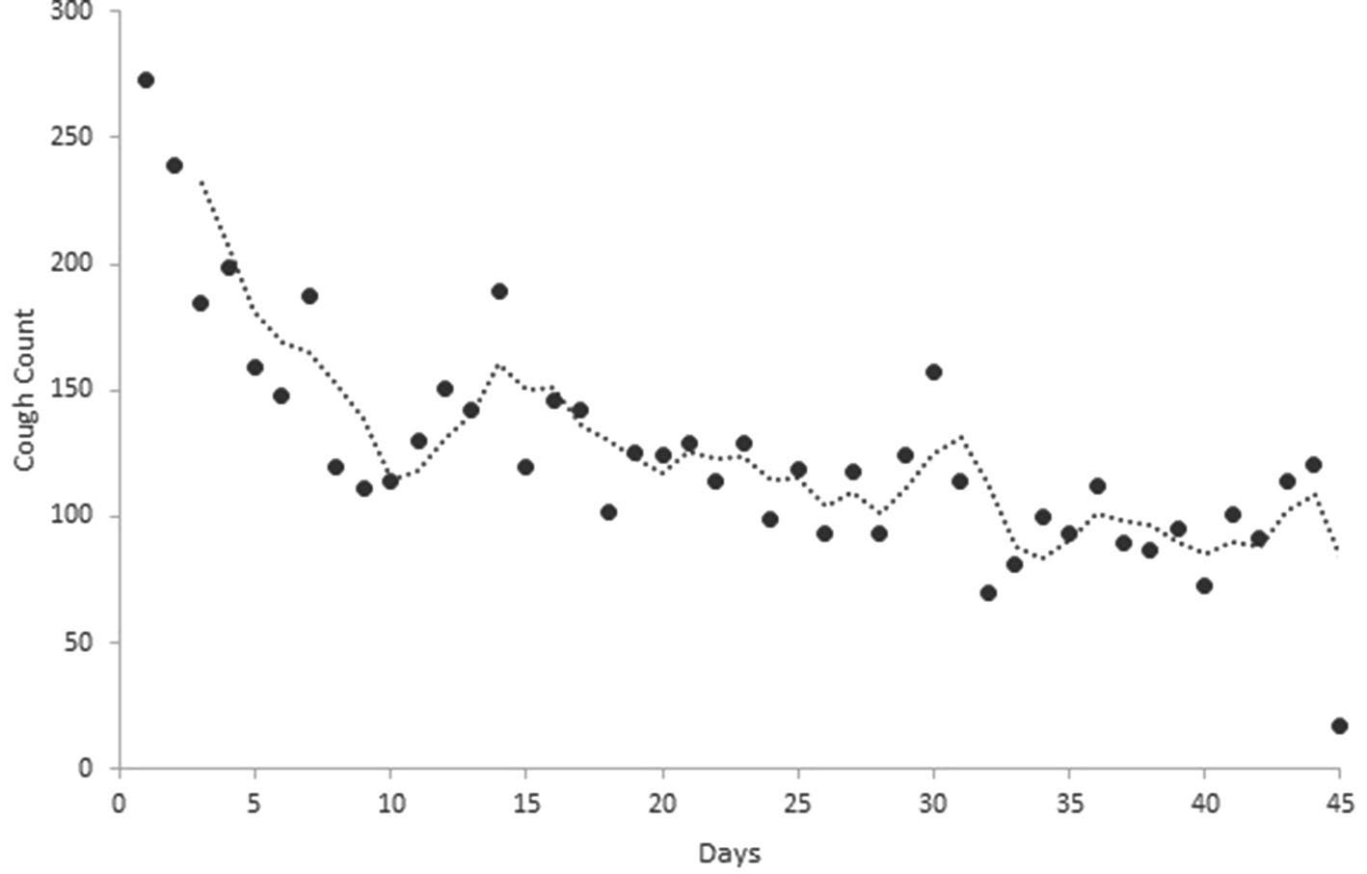
Mean daily cough count of all study participants (*circles*) and the 3-day rolling mean trend line (*dotted line*) (*n* = 16)

Daily cough counts were determined for individual patients (an example of an individual patient’s cough monitoring data is illustrated in Fig. 2). Mean values were calculated for the study population. Mean daily cough count fell significantly during the first 9 days from a mean ± SEM of 272.7 ± 54.5 on day 1 to 110.9 ± 26.3 on day 9 (*p* = 0.006). The mean daily cough count then appeared to plateau demonstrating day-to-day variation with a gradual declining trend to day 45 (Fig. 3).

### Correlation Between Continuous Cough Monitoring and the Hybrid HACC/LCM Cough Monitor

**Fig. 4:**
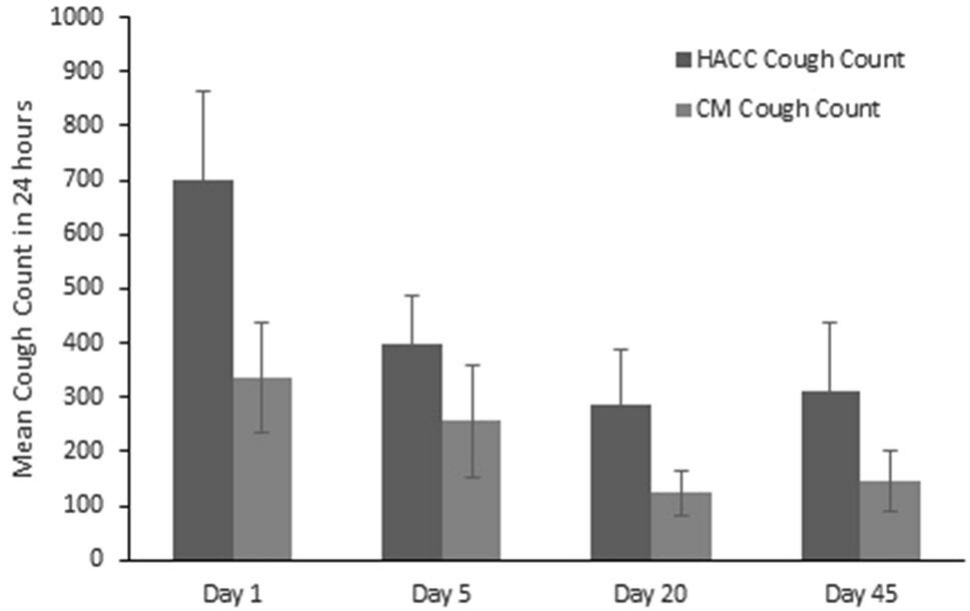
Mean cough count using HACC/LCM system and time-aligned mean cough count using the continuous monitoring (CM) system on days 1, 5, 20 and 45 following hospital discharge

**Fig. 5:**
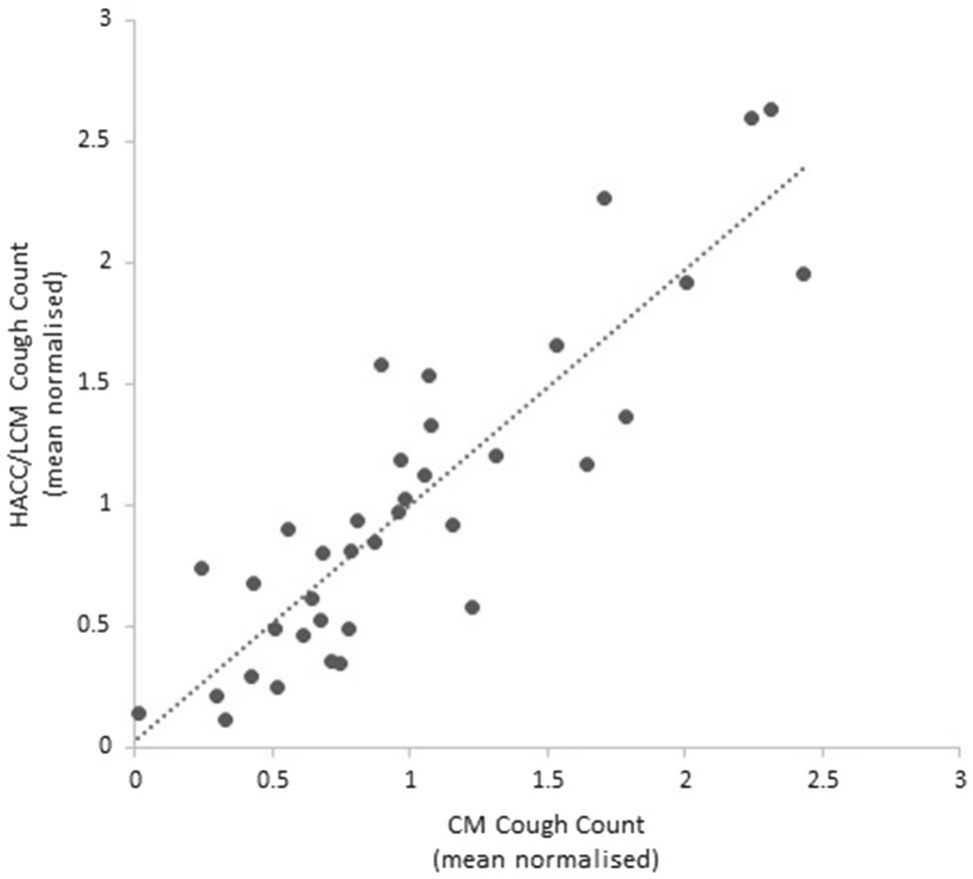
Correlation between mean normalised HACC/LCM cough count on days 1, 5, 20 and 45 and time-aligned mean normalised continuous monitoring (CM) system cough counts

The absolute mean daily cough count was significantly lower using the continuous monitoring system compared with the validated HACC/LCM system (Fig. 4, *p* = 0.005). There was a strong correlation between the mean normalised cough counts generated by the two cough monitoring systems (*r* = 0.88, *p* < 0.0001) (Fig. 5).

The mean ± SEM sensitivity of the continuous cough monitoring system was 50.7 ± 20.22% compared to the HACC/LCM system. We observed a considerable degree of interpatient variability in sensitivity; however, the intrapatient correlation was strong. There was no significant temporal change in sensitivity and specificity of the monitors throughout the study.

### Correlation Between Daily Cough Count and Symptoms

The subjective cough score decreased significantly during the first 7 days of monitoring (mean ± SEM cough score 2.56 ± 0.53 on day 1 and 1.92 ± 0.35 on day 7, *p* = 0.001). However, subjective cough scores only moderately correlated with objective daily cough counts (median *r* = 0.46 IQR 0.36–0.55). Objective cough counts showed the greater degree of change during recovery but subjective counts returned to convalescent levels sooner. Objective cough count did not correlate with other daily reported symptoms including shortness of breath (median *r* = 0.22 IQR −00.5 to 0.32), chest tightness (median *r* = 0.16 IQR 00.06 to 0.38), disturbed sleep (median *r* = 0.13 IQR −0.12 to 0.25), or sputum colour (median *r* = 0.04 IQR −0.13 to 0.25).

## Discussion

Current COPD monitoring strategies use a variety of symptom scores and measurement of basic physiological metrics including pulse rate, oxygen saturation and home spirometry. A goal of telemonitoring in COPD is the early identification of exacerbations facilitating timely intervention. There have been attempts to develop new tools to identify exacerbations including symptom scores such as the EXACT-PRO [11]; however, their utility in telemonitoring remains unclear and systems that rely predominantly on reported symptoms suffer from their subjective nature with the risk of ceiling effects and questionnaire fatigue. Objective measures therefore remain important. However, it has been demonstrated that the analysis of the commonly measured variables rarely identifies a clear distinction between normality, technical error, isolated bad days as part of day-to-day variation and exacerbations [7]. It has been reported that a composite score reflecting mean fall in oxygen saturation and rise in heart rate may aid in the identification of exacerbations over day-to-day variability [12], but this has not been confirmed in later studies [7]. Home spirometry has not been demonstrated to add additional useful information, and we have found poor compliance and poor performance confounding its utility [10]. A reliable objective measure that collects the data in a passive manner, not requiring regular patient involvement, is therefore desirable.

In this study, we demonstrate that it is possible to monitor trends in cough frequency using a device that records ambient sound within a patient’s home. Although the ability of the continuous monitoring system to identify absolute cough counts was significantly lower than that of the hybrid HACC/LCM cough monitor, it was clearly capable of identifying trends in cough frequency. A reduction in cough frequency over the first 9–10 days of AE-COPD convalescence with a gradual decline up until day 45 was detected. Although there was some interpatient variability in terms of cough detection sensitivity, the intrapatient correlation was generally very high. Thus, trends could be reliably identified.

Similar to previous reports, there was only a moderate correlation between the patient-reported symptom of cough and objective cough monitoring [13]. Although the subjective cough score decreased during the first 7 days of AE-COPD convalescence, the magnitude of change in mean score was less than 1 full point on the Likert scale, making it difficult to differentiate meaningful change from day-to-day variation for an individual patient. The magnitude of change in mean cough frequency between day 1 and day 7 was 119 coughs per 24 h representing a 40% reduction in cough frequency.

Objective measurement of cough frequency in COPD patients represents a novel metric for disease monitoring with the potential to enhance the capability of existing telemonitoring algorithms to detect true deterioration in clinical state. We believe that the utility of this technology is in monitoring trends in an individual patient’s cough frequency rather than absolute cough counting.

This study is small with relatively few patients. The requirement for patients to report a cough at study entry introduces selection bias to those with a subjective increase in cough associated with their AE-COPD. The utility of cough monitoring in COPD patients without a prominent cough at the time of exacerbation is therefore unknown. Recruitment to the study was challenging because potential participants were put off by the privacy implications of continuous recording of sound within their home. Real-time, automated analysis in order to negate the need for continuous sound recording would improve acceptability to patients. It is possible that patients may have consciously modified their cough frequency because of an awareness of being monitored. However, we feel that this is very unlikely given the unobtrusive and continuous nature of the monitoring system and long-term duration. The strong correlation between day and night cough counts that we have previously reported using the HACC/LCM system supports the validity of our data as any conscious modification of cough would be more likely during the day than at night [10]. For the purposes of analysing the change in objective cough frequency and subjective cough scores, missing data were replaced by the next available data point for that subject. This is likely to underestimate the significance of change over time and therefore we feel that the significant change observed in this study is valid.

Cough monitoring in this study is limited to AE-COPD convalescence and therefore we cannot evaluate the ability of the monitoring system to detect clinical change in the period preceding AE-COPD. Prospective studies of devices with inbuilt real-time analysis will be required to demonstrate the practical application of the technology in the home monitoring of COPD.

## Conclusion

Cough frequency declines significantly following AE-COPD and the reducing trend can be detected using continuous ambient sound recording and novel cough classifier software. Objective measurement of cough frequency in COPD patients has potential to enhance the capability of existing telemonitoring algorithms to detect true deterioration in clinical state.
